# Report of the Distribution Committee

**Published:** 1918-12-21

**Authors:** 


### KING EDWARD'S HOSPITAL FUND—(*continued*).

### REPORT OF THE DISTRIBUTION COMMITTEE.

1. The Governors and General Council have this year fixed the sum available for distribution amongst the London Hospitals at £190,000, as against £181,000 in 1917, £162,500 in 1916, and £149,500 in 1913, the last year before the war.

2. The number of hospitals applying for grants is 106.

3. Of the total amount distributed, £160,250 has been applied in the form of grants to maintenance. This is an increase of £8,075 over the corresponding total in 1917, and of £16,825 over that of 1916.

4. The capital grants this year amount to £29,750, an increase of £925 as compared with 1917, and an increase of £10,675 as compared with 1916. Of this year's total, only £4,150 is in respect of schemes sanctioned during the war on the ground of exceptional urgency, while £10,100 is in reduction of capital liabilities incurred before the war. The remaining £15,500 has been earmarked for schemes of improvement, the execution of which had been deferred until after the war.

5. During the course of the war a large number of hospitals have prepared schemes of improvement or extension to be carried out after the return of peace, and have submitted their proposals either in principle or in detail to the Fund. During the first three years of the war the Distribution Committee, with the approval of the General Council, discouraged the issue of all public appeals in aid of schemes which were not of such urgency as to require their being put in hand at once. Their reasons for this were: (*a*) that it was undesirable that appeals for future objects should compete with appeals for purposes which were urgently necessary during the period of national stress; (*b*) that it was undesirable to accumulate funds earmarked for future building when it was impossible to foresee the circumstances which would obtain after the war.

At the distribution of 1917, however, the committee for the first time recommended some capital grants in aid of the most urgent of the deferred schemes; and they have continued the same policy this year.

In April 1918 they announced their readiness to consider proposals for the immediate issue of appeals for post-war schemes, on certain conditions, one of which was: "that wherever the carrying out of the scheme would involve increased maintenance expenditure it should be clearly stated in the appeal that contributions received in response thereto should be available either for building, or for endowment in the event of circumstances rendering it inadvisable to expend the whole of the contributions on building."

6. The importance of ensuring, by some such means as this, that neither the Fund nor the individual hospitals are committed in advance to extensions on any fixed scale is shown by the fact that, if all the schemes which have come to the notice of the Fund were to be completed, the number of beds to be maintained by the voluntary hospitals of London would be increased, as compared with those maintained in 1913, by not less than 1,750.

The committee at the present stage do not desire to express any opinion as to whether this number of additional beds is or is not required. Some of the schemes have been launched without the views of the Fund having been sought. But in order to be in a position to consider the whole question from the point of view of the total needs of the population, the geographical distribution of the proposed increases, and the financial problem of future maintenance, the committee have decided to invite all the hospitals concerned to furnish them with the latest particulars of their building schemes, whether previously submitted in detail to the Fund or not.

In the meantime they have not considered at this year's distribution any applications for grants in aid of the provision of additional beds, except in the special case mentioned in the next paragraph.

7. The committee include in their awards this year a special grant of £5,000 towards the Woolwich and District War Memorial Hospital Building Fund.

### KING EDWARD'S HOSPITAL FUND—(*continued*).

It is not the practice of the Fund to assist in the building of new hospitals or to entertain applications from hospitals which have not been in existence in a properly constituted form for a period of three years. In 1909, however, the Distribution Committee announced their desire to see a representative movement started in Woolwich for the provision of an efficient general hospital of fair size, and stated that in consideration of the special local circumstances they would be prepared to recommend the General Council to support such a movement by a modification in this instance of the rule as to new hospitals, so as to allow of a conditional building grant.

The proposed War Memorial Hospital in Woolwich is the outcome of such a representative movement, and the Distribution Committee recommend that a grant of £5,000 should be made this year as a first contribution towards the erection of a new hospital in accordance with a scheme to be submitted to the Fund in due course.

8. The Committee have pleasure in reporting the receipt of proposals for the amalgamation of University College Hospital with the Royal Ear Hospital, whereby the latter, which has been closed during the war, will be re-opened as a special department of the former. The Committee recommend that the balance of £500 remaining unallocated out of the £10,500 set aside for the amalgamation of throat hospitals should be given to this scheme, and that a further grant of £500 be made to the same object out of this year's distribution.

By this amalgamation, following on that of the Hospital for Diseases of the Throat, Golden Square, with the London Throat Hospital, the number of ear, nose, and throat hospitals which formerly applied to the Fund will be reduced from five to three, of which only two are now on the books of the Fund, and the Committee do not now ask for any powers in respect of the sums conditionally promised for further amalgamations.

9. The Committee have as usual taken into consideration the returns made by the hospitals relating to the treatment of naval and military patients during the previous year, and also during the first nine months of the current year.

10. The following grants in aid of schemes of building expenditure which have been deferred during the war are recommended for renewal: Victoria Hospital (1916), £200 to heating; Hospital and Home for Sick Children, Sydenham (1915), £75 to central heating; Queen Charlotte's Lying-in Hospital (1913), £1,000 to new out-patient department; St. Mary's Hospital for Women and Children, Plaistow (1913), £1,500 to new nurses' home and out-patient department.

11. In dealing with questions of cost per bed the Committee have, as before, given full weight to the inevitable increases of cost which result from the rise in prices and in wages, the difficulty of obtaining skilled service, and other causes common to all hospitals, and have only taken into account cases where the cost is high in comparison with other hospitals or where there has been a disproportionate rise.

12. The Committee are again greatly indebted to the visitors for their very valuable reports. The services of the visitors are the more appreciated at the present time because of the difficulty under which the work of inspection is carried on by medical men and laymen who are already fully occupied by extra duties arising out of the war.

13. The details of the awards to the various hospitals are appended.

For the Committee, W. S. Church, Chairman.

December 1918.

